# Consumer-Based Activity Trackers as a Tool for Physical Activity Monitoring in Epidemiological Studies During the COVID-19 Pandemic: Development and Usability Study

**DOI:** 10.2196/23806

**Published:** 2021-04-23

**Authors:** André Henriksen, Erlend Johannessen, Gunnar Hartvigsen, Sameline Grimsgaard, Laila Arnesdatter Hopstock

**Affiliations:** 1 Department of Community Medicine UiT The Arctic University of Norway Tromsø Norway; 2 Department of Computer Science UiT The Arctic University of Norway Tromsø Norway

**Keywords:** COVID-19, energy expenditure, steps, smart watch, fitness tracker, actigraphy, public health, lockdown, SARS-CoV-2, pandemic, wearables

## Abstract

**Background:**

Consumer-based physical activity trackers have increased in popularity. The widespread use of these devices and the long-term nature of the recorded data provides a valuable source of physical activity data for epidemiological research. The challenges include the large heterogeneity between activity tracker models in terms of available data types, the accuracy of recorded data, and how this data can be shared between different providers and third-party systems.

**Objective:**

The aim of this study is to develop a system to record data on physical activity from different providers of consumer-based activity trackers and to examine its usability as a tool for physical activity monitoring in epidemiological research. The longitudinal nature of the data and the concurrent pandemic outbreak allowed us to show how the system can be used for surveillance of physical activity levels before, during, and after a COVID-19 lockdown.

**Methods:**

We developed a system (mSpider) for automatic recording of data on physical activity from participants wearing activity trackers from Apple, Fitbit, Garmin, Oura, Polar, Samsung, and Withings, as well as trackers storing data in Google Fit and Apple Health. To test the system throughout development, we recruited 35 volunteers to wear a provided activity tracker from early 2019 and onward. In addition, we recruited 113 participants with privately owned activity trackers worn before, during, and after the COVID-19 lockdown in Norway. We examined monthly changes in the number of steps, minutes of moderate-to-vigorous physical activity, and activity energy expenditure between 2019 and 2020 using bar plots and two-sided paired sample *t* tests and Wilcoxon signed-rank tests.

**Results:**

Compared to March 2019, there was a significant reduction in mean step count and mean activity energy expenditure during the March 2020 lockdown period. The reduction in steps and activity energy expenditure was temporary, and the following monthly comparisons showed no significant change between 2019 and 2020. A small significant increase in moderate-to-vigorous physical activity was observed for several monthly comparisons after the lockdown period and when comparing March-December 2019 with March-December 2020.

**Conclusions:**

mSpider is a working prototype currently able to record physical activity data from providers of consumer-based activity trackers. The system was successfully used to examine changes in physical activity levels during the COVID-19 period.

## Introduction

Physical activity is an important lifestyle factor [1] associated with a range of health outcomes [2]. Physical activity questionnaires and accelerometers are widely used to measure physical activity in epidemiological studies. The widespread use of advanced consumer-based activity trackers with a growing list of sensors and capabilities [3] has increased the use of activity trackers for research purposes [4]. New activity trackers are continuously released, and although the validity of most currently used activity trackers is unknown, a recent systematic review showed that interdevice reliability is often very strong [5].

This unique source of longitudinal physical activity recordings can be used to measure change in physical activity over time. It is therefore of interest to develop a system for automatic and continuous recording of physical activity data from available providers. This system can be used in a range of different research projects, including as a tool for physical activity surveillance.

The disease outbreak of COVID-19 (SARS-CoV-2) started in China December 2019, spread rapidly, and became a global pandemic. The first case of COVID-19 in Norway was confirmed February 26, 2020. On March 12, the Norwegian government implemented a lockdown of all schools, kindergartens, universities, high schools, gyms, etc, with additional restrictions in the following days. Although a national curfew was not instigated, people were encouraged to stay at home if possible. The most restrictive measures were gradually lifted from the end of April throughout May 2020. Less intrusive social distancing restrictions were gradually reintroduced throughout the Autumn, but no second lockdown was instigated in 2020.

In addition to the societal cost of the COVID-19 pandemic [6], physical inactivity during the lockdown and failing to revert to normal physical activity routines after the lockdown may cause health harm [7].

The aim of this study was to develop a system for automatic continuous recording of physical activity data from a range of consumer-based activity tracker providers and to examine its usability as a tool for physical activity monitoring in epidemiological research. The longitudinal nature of the data, and concurrent pandemic allowed us to examine how this system could be used to monitor change in physical activity before, during, and after the COVID-19 lockdown.

## Methods

### System Architecture

We designed and developed an experimental system, mSpider, intended for automatic and continuous recording of physical activity data using consumer-based activity trackers. The system collects data on physical activity, energy expenditure, pulse, sleep, and related variables over an extended period and from a range of providers and activity tracker models.

**Figure 1:**
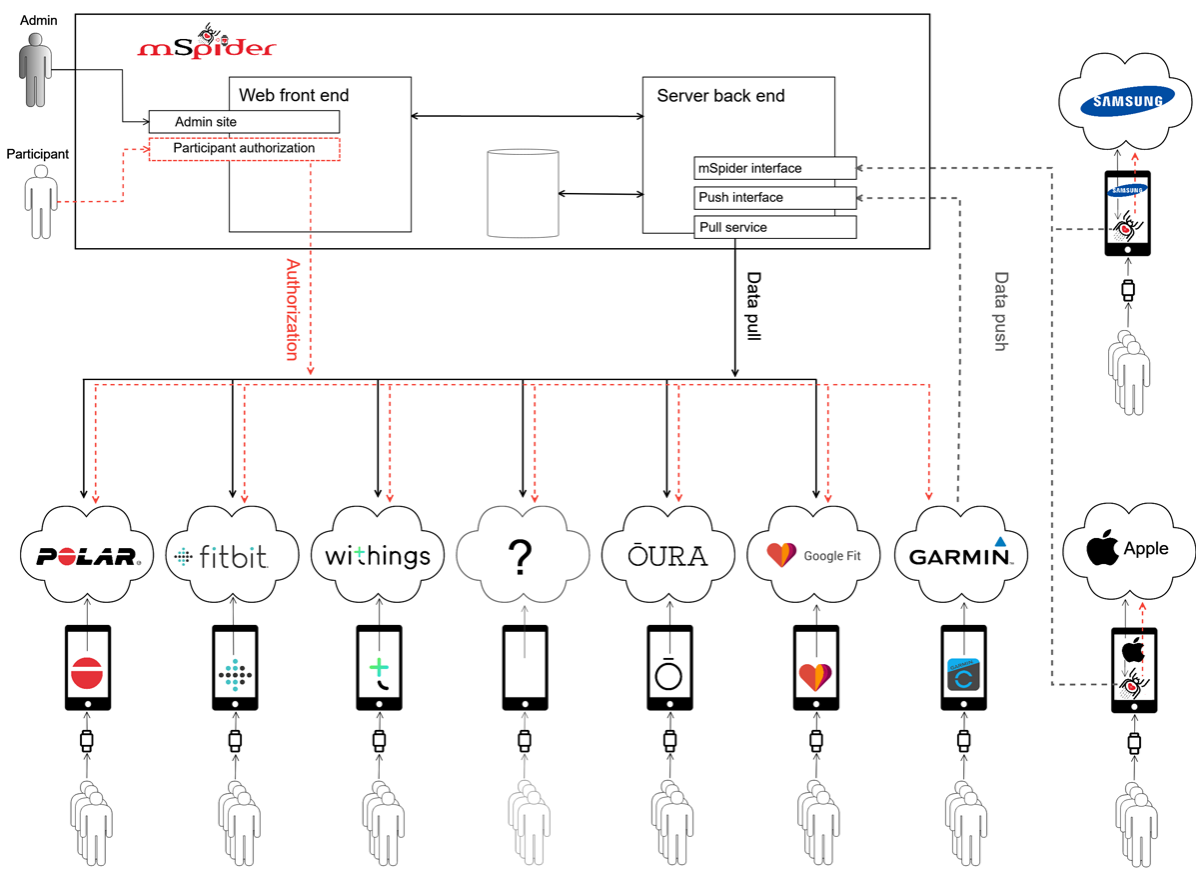
mSpider system architectural overview.

The system consists of three modules (see Figure 1): (1) the *web front end*, (2) *the server back end*, and (3) the *mobile app*. The *web front end* is used for managing surveys and to facilitate participant authorization when granting access to their activity tracker data. The *server back end* stores participant authorization access information, handles data transfer between mSpider and the cloud storages of supported providers, and stores downloaded activity tracker data. The *mobile app* further facilitates authorization and data transfer for providers where communication cannot be performed directly between the server back end and the provider cloud storage (eg, Samsung and Apple activity trackers). For these providers, communication is performed through the provider mobile app and uploaded to the mSpider server back end via the mSpider mobile app.

Figure 1 gives an architectural overview of the mSpider system, which providers are supported, and communications paths between systems. Red dashed lines indicate communication paths for participant authorization. To share data, users of Samsung and Apple activity trackers must install the mSpider mobile app and initiate authorization through this app via the provider mobile app. All other supported providers initiate authorization via the web front end, using open authorization, and participants are not required to install the mSpider app. Black solid lines between the *server back end* and external systems show providers where the server back end initiates a pull request to fetch data directly from the provider cloud storage, after access is granted by the participant. Gray dashed lines show providers where data transfer is initiated at the provider side (eg, Garmin) using a push request to provider-specific interfaces on the server back end. Data collected by the mSpider mobile app are also pushed to the mSpider server back end.

### Authorization

Participants authorize the mSpider system and grant access to their activity tracker data using OAuth. OAuth is an open protocol for allowing users to securely authorize data sharing between systems, without sharing user log-on credentials [8].

Pull requests from the mSpider system to external application programming interfaces (APIs; eg, Fitbit Web API) contain a *client identifier* and *client secret*, identifying mSpider as an authorized app for data retrieval. These credentials are given by the external system (ie, providers) upon successful registration of the mSpider app with each provider.

In addition, a *token identifier* and *token secret* are provided by the external system when an activity tracker user registers to participate in a study. Tokens are used to identify participants in future pull requests to the provider cloud storage (or push request from the provider). No directly identifiable information is transferred between the provider systems and the mSpider system. All communication is encrypted through the secure socket layer protocol (ie, HTTPS).

### Provider Support and Available Data Types

We developed support for activity trackers from Fitbit, Polar, Garmin, Withings, Samsung, Oura, and Apple, as well as providers that store data in Google Fit or Apple Health open health clouds (eg, Huawei). Except Samsung and Apple, supported providers offers a representational state transfer (REST) API web service. The REST software architectural style provides a set of constraints for distributed systems [9] and is a style commonly used when developing web services. A RESTful API (ie, an API using http requests; eg, GET, POST) uses a stateless architecture where the necessary information, including participant identification (ie, tokens), is transferred with the request. To access data from providers not supporting a REST API, the mSpider mobile app was developed using provider-specific software development kits (SDKs), which give access to activity tracker data via the provider-specific mobile app. Table 1 gives an overview of providers and which API or SDK we used to access data.

Each provider offers a different set of data types through their API or SDK. Steps is the only variable supported by all providers. Table 2 gives a list of available variables relevant for this study for each provider and how we used these variables to define valid days (ie, days where activity tracker wear time was sufficient enough to be included in daily physical activity analysis). A complete list of available variables can be found in the provider documentations (Table 1).

**Table 1:** Provider data access details.

Provider documentation	API^a^/SDK^b^	Version
Apple [10]	HealthKit	6.4
Fitbit [11]	Web API	1/1.2
Garmin (must register to gain access)	Health API	2.9.7
Google [12]	Fit API	1
Oura [13]	Cloud API	1
Polar [14]	AccessLink API	3.36.0
Samsung [15]	Health SDK	1.4.0
Withings [16]	Data API	2.0

^a^API: application programming interface.

^b^SDK: software development kit.

**Table 2:** Available variables by provider.

Provider	Variables	Valid day calculation
Apple	Steps, AEE^a^, REE^b^, sleep	Step>150
Fitbit	Steps, TEE^c^, AEE, LPA^d^, MPA^e^, VPA^f^, sleep	Step>150
Garmin	Steps, TEE, AEE, MPA, VPA	(Sleep + sedentary time + LPA + MPA + VPA) >10 hours
Google Fit	Steps, TEE	Step>150
Oura	Steps, TEE, AEE, sedentary time, LPA, MPA, VPA, nonwear time	Step>150
Polar	Steps, TEE, AEE, sedentary time, LPA, MPA, VPA, sleep	Nonwear time<14 hours
Samsung	Steps, AEE, sleep	(Sleep + sedentary time + LPA + MPA + VPA) >10 hours
Withings	Steps, TEE, AEE, LPA, MPA, VPA, sleep	Step>150

^a^AEE: activity energy expenditure.

^b^REE: resting energy expenditure.

^c^TEE: total energy expenditure.

^d^LPA: light physical activity.

^e^MPA: moderate physical activity.

^f^VPA: vigorous physical activity.

### Recruitment of Volunteer and Study Participants

#### Volunteers (Development Phase)

To test the system during development and increase the likelihood of long-term recording, we used convenience sampling to recruit 35 volunteers with the following inclusion criteria: 18 years or older, willing to wear a provided activity tracker for an extended period, and willing to share collected physical activity data. Data from these volunteers were used for system development and debugging purposes only and were not included in the longitudinal analysis of physical activity.

Volunteers were recruited during the development phase (from February 2019 to August 2020) and equipped with an activity tracker from Apple, Fitbit, Garmin, Huawei, Oura, Polar, Samsung, or Withings. Two volunteers also shared mobile phone–collected physical activity data stored in Google Fit. One volunteer withdrew after a few days, and two volunteers withdrew after a few months. We gave no instructions on activity tracker use, except giving instructions on how to initiate automatic data sharing with the mSpider system. Volunteers were given written and oral information about the mSpider system and informed that all collected data would be stored at the activity tracker provider’s cloud storage. All volunteers signed informed consent.

#### Study Participants (Physical Activity Study)

Through online news media advertisement, we recruited 130 people with privately owned activity trackers, worn before, during, and after the Norwegian COVID-19 lockdown. Inclusion criteria were owned an activity tracker from Garmin, Fitbit, Withings, or Oura and willing to share physical activity data. Recruitment was conducted in October 2020. Participants received an email invitation with a letter of information and instructions on how to grant access to the mSpider system. Participants gave informed consent by actively granting access to their data.

### Privacy

The 35 volunteers who received an activity tracker were required to register a user account at the activity tracker provider. Although the mSpider system only accessed nonidentifiable information, volunteers were informed that, by registration of a provider account, all data collected by the activity tracker would be uploaded to the provider cloud storage, including potential identifiable information (eg, GPS data).

The 130 study participants for analysis of activity tracker data already owned an activity tracker and thus already had a provider user account. After downloading the relevant data, we removed user tokens from the mSpider database and thus stored data anonymously.

### Data Collection

Daily estimates for steps, activity energy expenditure, moderate physical activity, and vigorous physical activity were downloaded from study participants. A variable for moderate-to-vigorous physical activity (MVPA) was created by combining moderate physical activity and vigorous physical activity for participants where these variables were available. We further downloaded light physical activity, sedentary time, sleep duration, and nonwear time, to be used for activity tracker wear time estimates. Data download was limited to days between January 1, 2019, and December 31, 2020. Only data from study participants (ie, not from volunteers) were included in the physical activity analyses.

Only days where the activity tracker was worn for at least 10 hours were labeled as *valid days* [17]. As this was not possible for all providers (Table 2), days with less than 150 recorded steps were excluded. After data download was completed, we removed the connection between the user’s provider and the mSpider tool by deleting user tokens. All data on physical activity was thus stored anonymously. An anonymous online questionnaire was sent to participants to collect self-reported data on sex, age, height, and weight.

### Statistical Analysis

Participant characteristics from the online questionnaire are presented as means, SDs, and ranges. For each participant, we used valid days to create monthly and yearly averages for steps per day (steps/day), activity energy expenditure in kilocalories per day (kcal/day), and MVPA in minutes per day (minutes/day) for 2019 and 2020. March 2020 was divided into two periods (up to and after March 12; ie, the lockdown date). For each variable we compared the following: 2019 (March-December) with 2020 (March-December); March 2019 with March 1-12, 2020; March 2019 with March 13-31, 2020; April 2019 with April 2020, May 2019 with May 2020, etc; March 2020, 1-12 with 13-31.

We created bar plots to visualize differences between time periods. Normality was checked using histograms. We used two-sided paired sample *t* test or two-sided paired Wilcoxon signed-rank test, depending on normality, to test differences in physical activity between time periods. Differences between periods were analyzed by only including participants with data from both periods. Two-sided *P* values <.05 were considered statistically significant. Statistical analyses were performed using R version 4.0.3 (R Foundation for Statistical Computing).

### Ethical Approval

The Regional Committees for Medical and Health Research Ethics North (reference 164780) and the Norwegian Center for Research Data (reference 628485) reviewed the study.

## Results

### Participant Characteristics

Of the 130 recruited study participants, 14 did not respond to the following invitation email and three owned an unsupported activity tracker. A final sample of 113 participants were thus included in the analysis. Of the included participants, 106 completed the online questionnaire and provided their characteristics (Table 3).

Due to the anonymous nature of the data collection, we did not have access to information about participant’s activity tracker model, only their provider. Altogether, 39 participants used Fitbit activity trackers, and 74 participants used Garmin activity trackers. No participants owned a Withings or Oura activity tracker.

Both Fitbit and Garmin provide data on steps, MVPA, and activity energy expenditure. All 113 participants were thus included when generating monthly means for all three variables. Monthly means were calculated from 66.274 measurements (ie, valid person-days).

**Table 3:** Participant characteristics (n=106).

Variable	Value	Range
Height (cm), mean (SD)	173.5 (8.0)	158-194
Weight (kg), mean (SD)	76.0 (14.3)	53.5-147.0
BMI (kg/m^2^), mean (SD)	25.2 (4.0)	18.3-50.3
Age (years), mean (SD)	40.6 (10.6)	21-69
Females, n (%)	59 (56.2)	N/A^a^

^a^N/A: not applicable.

### Change in Physical Activity

On average, participants walked 797 fewer steps per day in March 13-31, 2020, compared to March 2019 (*P*=.02). Similarly, participants walked on average 913 fewer steps per day in March 13-31, 2020 (postlockdown), compared to March 1-12, 2020, (prelockdown; *P*<.001). The remaining step comparisons showed no differences.

Mean activity energy expenditure was 74 kcal/day lower in March 13-31, 2020, compared to March 2019 (*P*=.02). In addition, mean activity energy expenditure was 85 kcal/day lower in March 13-31, 2020 (postlockdown), compared to March 1-12, 2020, (prelockdown; *P*=.001). However, activity energy expenditure was on average 54 kcal/day higher in September 2020 compared to September 2019 (*P*=.02). The remaining activity energy expenditure comparisons showed no difference.

For MPVA, monthly comparisons showed a significant increase from 2019 to 2020 for May (*P*=.01; with a median difference of 8 minutes), September (*P*=.008; with a median difference of 3 minutes), October (*P*=.02; with a median difference of 5 minutes), and December (*P*=.04; with a median difference of 4 minutes), as well as the yearly comparison (*P*=.03; with a median difference of 4 minutes). The remaining MVPA comparisons showed no difference.

**Figure 2:**
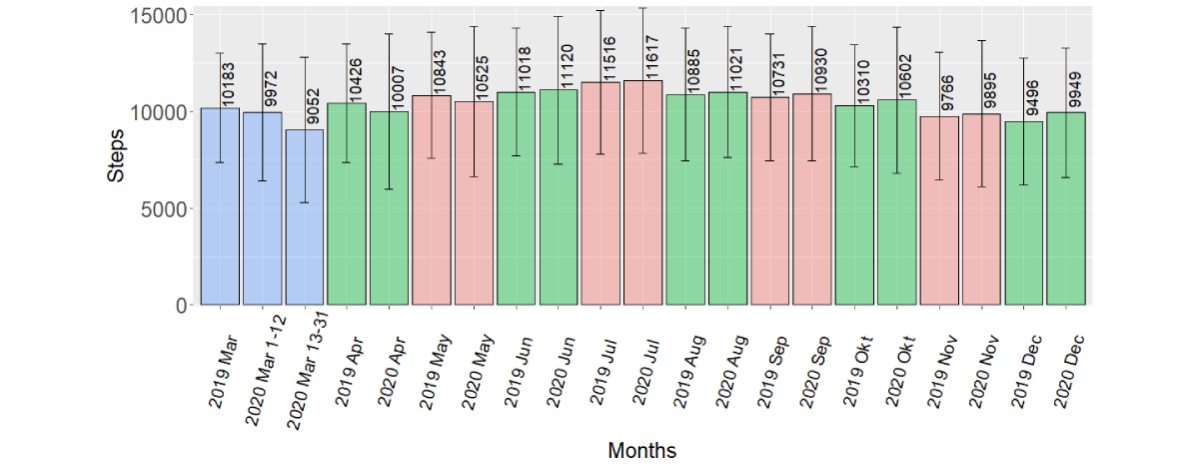
Bar plot of mean step count per day, by month, with SD (error bars).

**Figure 3:**
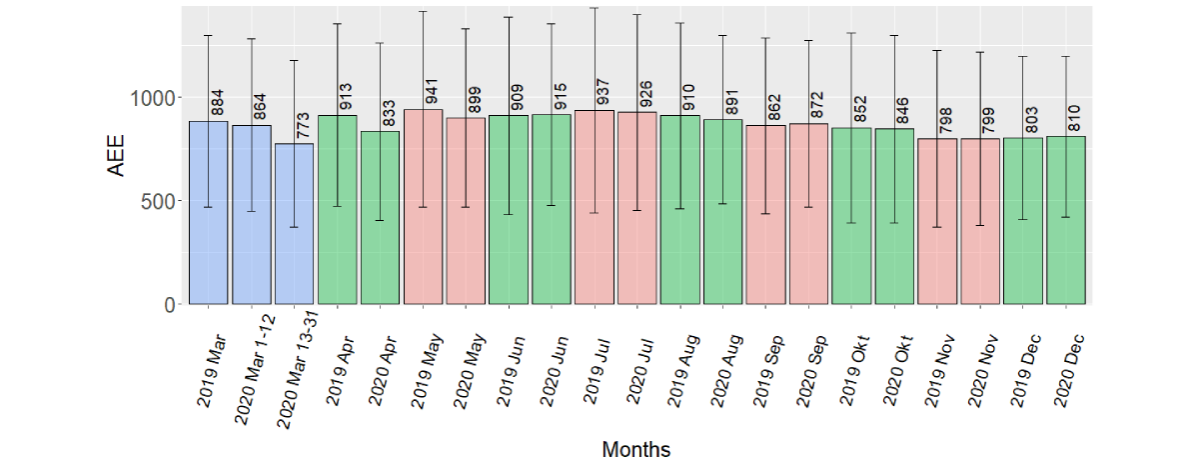
Bar plot of mean AEE per day, by month, with SD (error bars). AEE: activity energy expenditure.

**Figure 4:**
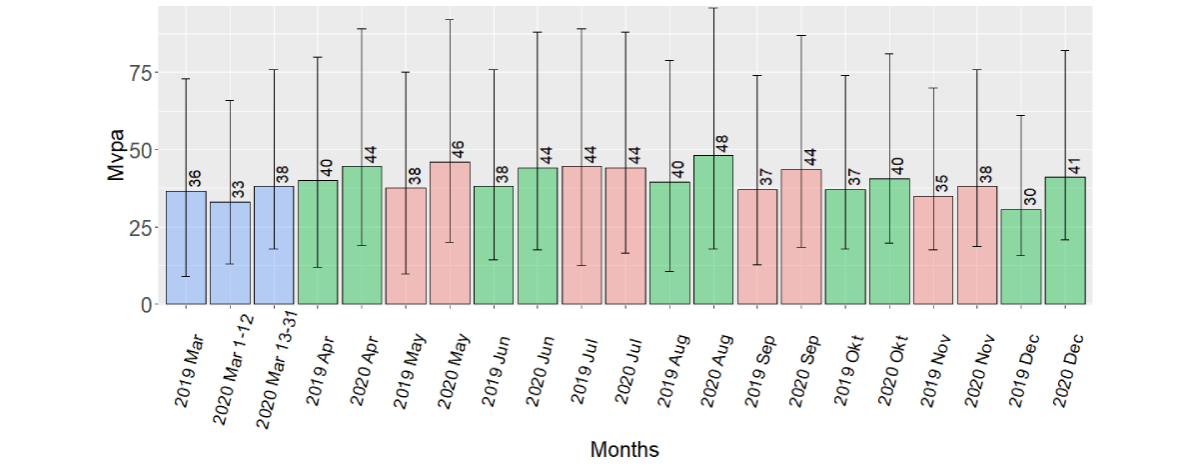
Bar plot of median minutes of MVPA per day, by month, with IQR (error bar). Mvpa: moderate-to-vigorous physical activity.

A summary of mean difference per day between periods for steps and activity energy expenditure, with 95% CIs and *P* values from each *t* test, is given in Table 4. The table also gives the median of the difference per day between periods for MVPA, with IQRs and *P* values from each Wilcoxon test. Because we used paired tests, analysis only include participants with data in both the preperiod and the postperiod, thus is based on data from 76 to 107 participants. Figure 2 and Figure 3 gives monthly mean step count and activity energy expenditure from March 2019 through December 2020. Figure 4 gives the median MVPA for the same periods.

**Table 4:** Difference per day between preperiods and postperiods.

Monthly comparison 2019-2020	Steps (steps/day), mean difference (95% CI)	*P* value^a^	AEE^b^ (kcal/day), mean difference (95% CI)	*P* value	MVPA^c^ (min/day), median (IQR)	*P* value
March-December	349 (–4 to 702)	.05	29 (–2 to 60)	.07	4 (–6 to 4)	.03
March 1-12^d^	28 (–608 to 664)	.93	21 (–40 to 82)	.49	–2 (–14 to –2)	.57
March 13-31^e^	–797 (–1468 to –126)	.02	–74 (–136 to –11)	.02	2 (–11 to 2)	.83
April	–123 (–850 to 605)	.74	–35 (–105 to 34)	.32	–1 (–15 to –1)	.81
May	53 (–586 to 692)	.87	2 (–59 to 64)	.94	8 (–6 to 8)	.01
June	301 (–276 to 878)	.30	45 (–7 to 97)	.09	4 (–10 to 4)	.07
July	442 (–232 to 1117)	.20	44 (–15 to 104)	.14	1 (–14 to 1)	.53
August	326 (–271 to 922)	.28	24 (–24 to 72)	.33	2 (–14 to 2)	.53
September	324 (–148 to 797)	.17	54 (8 to 100)	.02	3 (–7 to 3)	.008
October	361 (–290 to 1011)	.27	41 (–7 to 89)	.10	5 (–6 to 5)	.02
November	242 (–442 to 927)	.48	42 (–22 to 106)	.20	4 (–11 to 4)	.34
December	491 (–6 to 988)	.05	32 (–21 to 84)	.24	4 (–8 to 4)	.04

^a^*P* values from paired sample *t* test or paired Wilcoxon signed-rank test.

^b^AEE: activity energy expenditure.

^c^MVPA: moderate-to-vigorous physical activity.

^d^Comparing March 2019 with March 1-12, 2020.

^e^Comparing March 2019 with March 13-31, 2020.

## Discussion

### Principal Findings

In this study, the mSpider system was successfully used to download historic data on steps, activity energy expenditure, and MVPA from Garmin and Fitbit activity tracker users. The longitudinal data showed changes in physical activity during the COVID-19 pandemic.

Findings indicate a short-term reduction in steps and activity energy expenditure due to the COVID-19 lockdown but no reduction in MVPA. However, participants increased their level of MVPA the month after the lockdown period (ie, May 2020) and some months in the autumn of 2020 (ie, September, October, and December) compared to 2019.

### Comparison With Previous Work

Results in this study are supported by reports from providers of consumer-based activity trackers. Garmin have released a statement showing that users globally had a distinct decline in step count during the last 2 weeks of March 2020 and that the reduction in step counts was compensated by increase in other activities [18]. Withings have reported a temporary decline in step counts among users during national lockdowns [19]. Similarly, a study of UK adults using physical activity data recorded by a smartphone app showed a significant decrease in physical activity during the March 2020 UK national lockdown [20].

Google Trend analysis of community interest in physical activity during the COVID-19 outbreak and lockdown showed an increase in Google search rates on physical activity topics in Australia, the United Kingdom, and the United States [21]. A study among German athletes using activity tracker data showed that shorter and more vigorous exercise sessions replaced longer sessions [22].

These studies support our finding that, although restrictions confined people to their home, they found alternative ways to keep their habitual physical activity level. Conversely, based on online physical activity questionnaires, a study from Thailand did not show any increase in physical activity after the lockdown was lifted [23], and a study from Bangladesh showed a high prevalence of inactivity during lockdown [24].

In summary, activity tracker data from several vendors and groups of users including athletes and patients with a chronic disease have shown changes in physical activity levels and patterns during the COVID-19 pandemic, but findings vary between countries.

### mSpider as a Method for Data Collection on Physical Activity

The analysis of physical activity changes related to the COVID-19 pandemic period showed that the mSpider system can be a valuable tool for collection of long-term data on physical activity, including historical data and detecting changes in physical activity over time.

In this study, we used the proposed system to access data retrospectively from participants with privately owned activity trackers. Previously, we have successfully used the same technology for long-term prospective physical activity monitoring among participants in a lifestyle intervention study wearing a provided activity tracker for up to 1 year ([25,26] and Hopstock et al, unpublished data, 2020).

A system similar to mSpider, Remote Assessment of Disease and Relapses (RADAR)–base, was used by Sun et al [27], who observed change in daily steps during national lockdowns among participants with chronic disease equipped with a Fitbit tracker. RADAR-base is an open-source platform for collecting physical activity data from smartphones, Fitbit and Garmin activity trackers, and some research grade accelerometers [28]. RADAR-base uses similar technology as mSpider, but data collection is limited to only two providers of consumer-based activity trackers.

A study by Radin et al [29] successfully mapped historic Fitbit data (provided manually by Fitbit) to known influenza outbreaks. This also shows the potential for the proposed system as a tool for disease outbreak surveillance, where clusters of participants with a combination of physical activity reduction and elevated resting heart rate can be used to indicate disease outbreaks in an area.

The quality of accelerometer-based physical activity data is dependent on participant wear compliance. Future epidemiological research may benefit from the proposed system by facilitating long-term data recording, especially from younger adults who may be less compliant when wearing traditional accelerometers compared to older adults [30] but more likely to own and wear an activity tracker [31]. Long-term activity tracker data can thus add to and enrich accelerometer-based data collections, especially from younger participants.

In summary, we found the mSpider system to be an interesting supplement to current tools for physical activity monitoring in epidemiological studies. However, major challenges must be kept in mind. First, self-selected users of activity trackers are often more physically active compared to nonusers [31,32]. Second, the accuracy of different activity trackers can be highly variable and the choice of activity tracker will therefore affect reported performance [5,33,34]. At the population level, the system may perform better to detect change in physical activity over time than to estimate the absolute levels of physical activity.

### Strengths and Limitations

The major strength of this study is the long-term recording with up to 2 years of daily physical activity data per participant. This allowed for month-to-month comparisons between 2019 and 2020, thus taking potential seasonal differences in physical activity levels into account.

The study has limitations that can affect the study results. The participants were self-selected owners of physical activity trackers who were likely to be more physically active than the general population. A recent study by Anyan et al [35] investigating physical activity change during the Norwegian lockdown (using questionnaire data) found that 14% of participants reported a reduction, 22% reported an increase, and 64% reported no change in physical activity level. Therefore, there is a risk of selection bias in this study (ie, the sample may not be representative of the general population). Nevertheless, the observed changes in physical activity levels in this sample during the study period demonstrate the usefulness of the mSpider system. Further, due to anonymous data collection, we could not link participant characteristics to physical activity data to examine physical activity in strata of sex, age, or other characteristics (eg, activity tracker model).

### Conclusion

mSpider is a working prototype currently able to record physical activity data from several providers of consumer-based activity trackers. The system was successfully used to detect longitudinal changes in physical activity levels before, during, and after the Norwegian COVID-19 lockdown period in 2020. To our knowledge, this is the first study reporting change in physical activity caused by the COVID-19 lockdown in Norway using 2 years of objective consumer-based activity tracker data.

## Abbreviations

API: application programming interface
MVPA: moderate-to-vigorous physical activity
RADAR: Remote Assessment of Disease and Relapses
REST: representational state transfer
SDK: software development kit
